# Shared Genetics between Age at Menarche and Type 2 Diabetes Mellitus: Genome-Wide Genetic Correlation Study

**DOI:** 10.3390/biomedicines12010157

**Published:** 2024-01-11

**Authors:** Yuan-Fang Cheng, Cheng-Yi Yang, Meng-Che Tsai

**Affiliations:** 1 School of Medicine, College of Medicine, National Cheng Kung University, Tainan 70101, Taiwan; 2 Department of Statistics, College of Management, National Cheng Kung University, Tainan 70101, Taiwan; 3 Department of Pediatrics, National Cheng Kung University Hospital, College of Medicine, National Cheng Kung University, 138 Shengli Road, Tainan 70403, Taiwan; 4 Department of Genomic Medicine, National Cheng Kung University Hospital, College of Medicine, National Cheng Kung University, Tainan 70101, Taiwan; 5 Department of Medical Humanities and Social Medicine, College of Medicine, National Cheng Kung University, Tainan 70101, Taiwan

**Keywords:** age at menarche, type 2 diabetes mellitus, genome-wide association study, conditional false discovery rate, Taiwan Biobank

## Abstract

**Background:** Age at menarche (AAM) has been associated with type 2 diabetes mellitus (T2DM). However, little is known about their shared heritability. **Methods:** Our data comes from the Taiwan Biobank. Genome-wide association studies (GWASs) were conducted to identify single-nucleotide polymorphisms (SNPs) related to AAM-, T2DM-, and T2DM-related phenotypes, such as body fat percentage (BFP), fasting blood glucose (FBG), and hemoglobin A1C (HbA_1C_). Further, the conditional false discovery rate (cFDR) method was applied to examine the shared genetic signals. **Results:** Conditioning on AAM, Quantile-quantile plots showed an earlier departure from the diagonal line among SNPs associated with BFP and FBG, indicating pleiotropic enrichments among AAM and these traits. Further, the cFDR analysis found 39 independent pleiotropic loci that may underlie the AAM-T2DM association. Among them, *FN3KRP* rs1046896 (cFDR = 6.84 × 10^−49^), *CDKAL1* rs2206734 (cFDR = 6.48 × 10^−10^), *B3GNTL1* rs58431774 (cFDR = 2.95 × 10^−10^), *G6PC2* rs1402837 (cFDR = 1.82 × 10^−8^), and *KCNQ1* rs60808706 (cFDR = 9.49 × 10^−8^) were highlighted for their significant genetic enrichment. The protein–protein interaction analysis revealed a significantly enriched network among novel discovered genes that were mostly found to be involved in the insulin and glucagon signaling pathways. **Conclusions:** Our study highlights potential pleiotropic effects across AAM and T2DM. This may shed light on identifying the genetic causes of T2DM.

## 1. Introduction

According to the International Diabetes Federation, the global number of adults living with type 2 diabetes mellitus (T2DM) is predicted to rise to 643 million by 2030 and 783 million by 2045 [1]. Moreover, Taiwan has the highest prevalence of diabetes among Asian countries, and the prevalence is still rapidly rising by 25,000 people per year [2]. T2DM, with its occurrence mainly due to excessive body adiposity and insulin resistance, can lead to severe chronic complications, including cardiovascular, central and peripheral nervous, and immune disorders. Studies of twins have shown that genetics plays an essential role in the development of T2DM [3]. According to prior studies, several important genes have been indicated, including *CAPN10* (calpain 10), *PPARG* (peroxisome proliferator-activated receptor gamma), *KCNJ11* (potassium inwardly rectifying channel subfamily J member 11), *TCF7L2* (transcription factor 7-like 2), *KCNQ1* (potassium voltage-gated channel subfamily Q member 1), and *MTNRB1* (melatonin receptor B1) [4].

Early age at menarche (AAM) is usually defined as menarche before the age of 12 years. Over the past 30 years, AAM has decreased in Taiwanese children, with a rate of decline of approximately 0.43 years per decade [5]. Early menarche has been associated with physical and psychosocial problems, including anxiety/depression, substance use, suicidal behaviors, early fusion of epiphyseal growth plates, cardiovascular diseases, and metabolic syndromes [6]. AAM is a hereditary trait, but its known genetic determinants only explain part of the variance [7].

Early AAM and T2DM are highly heritable traits with rising prevalence, and both may adversely affect healthy life outcomes [8,9]. Studies have revealed an association between AAM and the risk of T2DM [10], as women with early menarche have a higher risk of T2DM in adulthood. Although excessive adiposity is assumed to be a common driver, the two traits may share genetic loci, but their shared heritability remains unclear. Therefore, a genome-wide association study (GWAS) is required to investigate whether AAM and T2DM have common heritable origins and further determine the underlying molecular pathways.

In this study, we aimed to identify shared genetic variants of AAM and T2DM and investigate the connection between the two traits using a nationally representative database. However, GWAS has underlying shortcomings, such as low genetic coverage, missing phenotypes, and low allele frequency. Quality control (QC) of genotypic and phenotypic data was conducted to reduce biases in GWAS. We hypothesize that genetic variants may show differential associations with T2DM on the condition of their associations with AAM.

## 2. Methods

### 2.1. Participants

Genotypic and phenotypic data were available for 88,347 participants from the Taiwan Biobank (TWB, https://www.twbiobank.org.tw, accessed on 27 April 2023). The TWB is a government-supported, prospective cohort study with a wide range of phenotype and genome data for the Taiwanese population [11]. TWB 2.0 axiom genome-wide array plate was designed for whole-genome genotyping. There are approximately 750,000 single-nucleotide polymorphisms (SNPs) in TWB 2.0 covering specific SNPs associated with diseases, drug metabolism, and drug response in the Taiwanese population [12]. Phenotypic data were obtained from questionnaires and measurements [13]. Participants were asked to report their age, education level, age at enrollment, AAM, diagnosis of diabetes, types of diabetes, and family history of diabetes in the questionnaire. Other T2DM-related phenotypes of interest, including body fat percentage (BFP), fasting blood glucose (FBG), and hemoglobin A1C (HbA_1C_), were obtained from measurements [14,15]. The present study was approved by the Institutional Review Board of National Cheng Kung University.

### 2.2. Quality Control Procedures

QC procedures were implemented using PLINK (https://www.cog-genomics.org/plink/, accessed on 10 May 2023) within R (4.3.0, Posit, PBC, Boston, MA, USA). The QC procedures (see Supplementary Table S1 and Figure S1 for details) can be divided into two parts: sample- and SNP-level filtering. Samples with a genotype missing rate ≥ 2% and missing phenotypes were excluded from the analysis. In addition, SNPs with a genotype missing rate ≥ 2%, minor allele frequency (MAF) < 0.01, and Hardy–Weinberg equilibrium (HWE) exact test *p*-value < 10^−6^ were filtered out. Finally, 53,224 Taiwanese female participants were included in the following analysis.

### 2.3. Statistical Analysis

As population genetic structures differ across ethnicities and the Taiwanese population is less representative in international data sources, we randomly split our samples into two datasets for the exploration of genetic association with AAM (trait 1; *N* = 26,612) and T2DM and relevant phenotypes (trait 2; *N* = 26,612). This method is referred to as the Mendelian randomization approach, which is used to minimize confounding effects. We tested between-group differences to ensure similarities in these two subsets regarding waistline, overall health, body mass index (BMI), BFP, hypertension [16], and years of education [17]. Principal components analysis (PCA) was performed to identify the structure in the distribution of genetic variation across the genetics of all the participants.

Two separate GWASs were conducted for AAM and T2DM traits using two exclusive subsets of data. GWAS for the AAM trait was adjusted for age at enrollment, BMI, and the principal components; GWAS for T2DM traits was adjusted for education level, BMI, and the principal components. Initially, we depicted conditional quantile-quantile (Q-Q) plots to visualize SNP enrichment for T2DM and relevant phenotypes given the different levels of association with AAM. A greater and earlier departure from the reference line (leftward shift) with a higher association with AAM indicates a more significant genetic overlap between AAM and T2DM traits. Further, we applied the conditional false discovery rate (cFDR) method, an extension of the conventional FDR incorporating information from the GWAS summary statistics of a conditional phenotype, to identify the shared genetic variants between AAM and T2DM. In this study, the cFDR is defined as the probability that an SNP is not associated with T2DM traits, given that the *p*-values for both AAM and T2DM traits are below the significance threshold, and its value indicates the level of pleiotropic enrichment. The cFDR analysis was implemented using an R package (https://github.com/jamesliley/cFDR-common-controls, accessed on 4 August 2023) [18]. The shared loci between the AAM and T2DM traits based on the cFDR analysis were visualized in a Manhattan plot. Since multiple significant association *p*-values in the same region were observed, a linkage disequilibrium (LD)-based clumping procedure was performed to estimate the number of independent loci, with sites less than 500 kb away from an index variant and with r^2^ larger than 0.1 assigned to a single clump. To identify the connectivity among the pleiotropic loci, we also used the Search Tool for the Retrieval of Interacting Genes/Proteins (STRING, https://string-db.org/, accessed on 21 August 2023) to build protein–protein interaction (PPI) networks.

## 3. Results

The average age of the entire study population was 50.9 years. Overall, the average AAM was 13.3 years, and a total of 4.57% reported being diagnosed with T2DM. The two randomly split datasets were similar in most baseline characteristics, age distribution, and risk of T2DM based on the ages of participants (see Supplementary Table S2 and Figure S2 for details).

**Figure 1:**
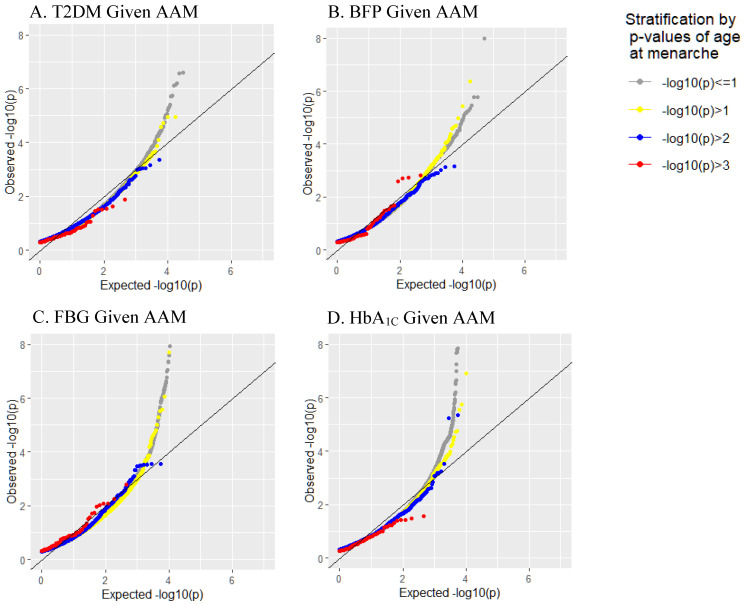
Conditional quantile–quantile plotsfor diabetic traits conditioned on *p*-values of AAM. The diagonal line indicates a null hypothesis. (Note: AAM, age at menarche; T2DM, type 2 diabetes mellitus; BFP, body fat percentage; FBG, fasting blood glucose; HbA_1C_, hemoglobin A1C).

**Figure 2:**
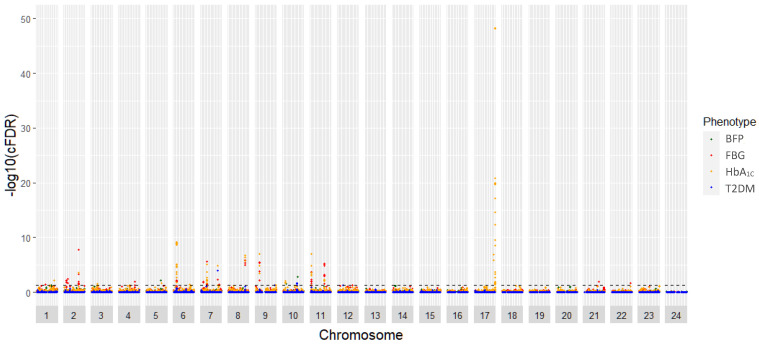
Conditional false discovery rate (cFDR) Manhattan plot of −log_10_ values for T2DM traits. The dashed line indicates a statistical significance threshold of a cFDR of less than 0.05. (Note: BFP, body fat percentage; FBG, fasting blood glucose; HbA_1C_, hemoglobin A1C; T2DM, type 2 diabetes mellitus).

We depicted conditional Q-Q plots showing SNP enrichment for T2DM traits given different levels of association with AAM (Figure 1). An earlier and greater departure from the reference line was well noted in the plots of BFP and FBG, indicating stronger pleiotropic enrichments being observed for these two traits conditioned on AAM. In contrast, the plots of T2DM diagnosis and HbA_1C_ levels given AAM showed low levels of pleiotropic enrichment. Based on the cFDR analysis, the shared loci between AAM and T2DM and relevant traits were visualized in a Manhattan plot (Figure 2). Using a cutoff of a cFDR less than 0.05, a total of 39 independent shared genetic loci were identified for the four T2DM traits after the clumping procedure (Table 1). The most significant one is *FN3KRP* rs1046896, which has the highest significant level of enrichment (cFDR = 6.84 × 10^−49^). In addition, *CDKAL1* rs2206734 (cFDR = 6.48 × 10^−10^), *B3GNTL1* rs58431774 (cFDR = 2.95 × 10^−10^), *G6PC2* rs1402837 (cFDR = 1.82 × 10^−8^), and *KCNQ1* rs60808706 (cFDR = 9.49 × 10^−8^) were highlighted for their significant genetic enrichment.

Further, to discover the connectivity among the pleiotropic loci, we built a PPI network using the STRING database, and the network was significantly enriched by showing that the nodes were not random and the observed number of edges was significant (see Supplementary Figure S3 for details). The top 2 Kyoto Encyclopedia of Genes and Genomes (KEGG) pathways were the insulin signaling pathway and glucagon signaling pathway (see Supplementary Table S3 for details).

## 4. Discussion

In this study, a genome-wide statistical approach was used to identify 39 shared loci between AAM and T2DM traits, with some reported in association with T2DM [19,20,21,22,23]. The conditional Q-Q plots showed pleiotropic enrichments for BFR and FBG conditioned on AAM, but the enrichment effect was less prominent for T2DM diagnosis and HbA_1C_ levels. This discrepancy may result from data sources and availability. The diagnosis was self-reported, and the medication history was unavailable in TWB. There was a possibility that T2DM was undiagnosed or diabetic types were misclassified by participants. Moreover, HbA_1C_ might reflect glycemic control corresponding to patients’ medication adherence rather than disease severity among patients with T2DM. Obtaining collateral reports from treating physicians or linked medication records may ameliorate data quality and accuracy.

Using the cFDR analysis, our study confirmed some novel enrichment loci in shared genetics between AAM and T2DM traits, such as rs1046896 (*FN3KRP*), rs2206734 (*CDKAL1*), rs58431774 (*B3GNTL1*), rs1402837 (*G6PC2*), and rs60808706 (*KCNQ1*). The SNP rs1046896 (*FN3KRP*) has the highest genetic enrichment level. *FN3KRP* (fructosamine-3-kinase-related protein) is involved in the deglycation of proteins modified via non-enzymatic glycation [24]. A high glucose concentration can result in the non-enzymatic oxidation of proteins via the reaction of glucose-6-phosphate and lysine residues. Proteins modified in this way become less active or functional. This reaction is referred to as the non-enzymatic glycation of proteins or the Maillard reaction. Hyperglycemia is a primary factor that promotes glycation, and the glycation end product forms on DNA, lipids, and proteins, where they represent pathophysiological modifications that precipitate dysfunction at a cellular and molecular level [25]. This further leads to several complications, such as blindness, heart disease, nerve damage, and kidney failure [26]. *FN3KRP* may result in the deglycation of proteins to restore their function.

*CDKAL1* (cyclin-dependent kinase 5 regulatory subunit associated protein 1 like 1) has been reported as a major pathogenesis-related protein for T2DM [27]. The function of *CDKAL1* has not been entirely determined. Research has shown that *CDKAL1* may be involved in beta cell dysfunction in the pancreas and the regulation of mitochondrial function in adipose tissue [28,29].

*B3GNTL1* (beta1,3-N-Acetylglucosaminyltransferase-Like Protein 1) protein is predicted to enable glycosyltransferase activity [30]. According to a recent study, analysis of the PPI network of the genes containing *B3GNTL1* reveals that, at the molecular level, there seemed to be interconnected factors that affect the progression of renal impairment among diabetic patients [31].

*G6PC2* (glucose-6-phosphatase catalytic subunit 2) catalyzes the hydrolysis of glucose-6-phosphate to produce glucose and inorganic phosphate. The gene is explicitly expressed in pancreatic islet beta cells. The mutation in *G6PC2* was found to have a lead role in the modulation of FBG levels, and, thus, it might increase the risk of T2DM [32]. In addition, previous GWAS showed linked polymorphisms in *G6PC2*, with variations in FBG and BFP [33].

*KCNQ1* was previously identified for its correlation with T2DM [34]. *KCNQ1* encodes a voltage-gated potassium channel required for the repolarization phase of the action potential of cardiac muscles [35], and the gene is also expressed in other tissues, including the brain, adipose tissue, and pancreas, as well as in the insulin-secreting cell line [36]. In addition to its well-studied association with T2DM, research has revealed a significant association with menopausal age [37].

The PPI analysis revealed that the whole network was significantly enriched. The proteins at the center of the PPI network were insulin (encoded by *INS*) and AKT serine/threonine kinase 1 (encoded by *AKT1*). Both are involved in glucose homeostasis and metabolism [38]. Moreover, the top two KEGG pathways were the insulin and glucagon signaling pathways. Insulin plays a critical role in multiple physiological processes, such as promoting glucose uptake, glycogen synthesis, lipogenesis, and protein synthesis. Reduced production of insulin and insulin resistance are the main causes of diabetes. Insulin is an anabolic peptide hormone secreted by pancreatic β cells acting through a receptor located in the membrane of target cells. The receptor activates a complex intracellular signaling network. The two main pathways of insulin signaling emanating from the insulin receptor-IRS node are the PI3K (phosphatidylinositol 3-kinase)/AKT (AKT serine/threonine kinase 1) pathway and the Raf/Ras/MEK (mitogen-activated protein kinase kinase)/MAPK (mitogen-activated protein kinase) pathway [39]. Glucagon is also an important regulator of glucose homeostasis, and it is secreted by pancreatic α cells. Once glucagon binds to a transmembrane receptor on target cells, it leads to adenylate cyclase activation and cAMP formation. The increase in intracellular cAMP levels activates PKA (protein kinase A), which phosphorylates CREB (cAMP response element binding protein), PFK-2 (phospho-fructokinase 2)/FBPase2 (fructose 2,6-bisphosphatase), pyruvate kinase, phosphorylase kinase, and glycogen synthase. Overall, glucagon increases gluconeogenesis and glycogenolysis and decreases glycolysis [40]. Most of the novel-discovered genes were also found to be involved in these pathways. For example, *CREB* encodes the protein that regulates the gene expression of the enzymes of gluconeogenesis, and it has recently been regarded as a targeting factor in diabetic treatment [41].

Our findings may have implications for clinical practices and directions for future research. Firstly, genetic screening for the shared loci between AAM and T2DM may inform lifestyle modification and glycemic monitoring among early-maturing women. In this sense, further exploration of the gene–lifestyle interaction effects on T2DM may help to identify high-risk groups targeted for advanced pharmacotherapy. Moreover, enriched pathways involving shared genetics may shed light on experimental research into T2DM mechanisms associated with early AAM, a critical step toward personalized diabetic prevention and medication strategies.

Despite its population size and representativeness strength, this study has several limitations. Firstly, the genetic association analysis was derived from and, thus, may be limited to the Taiwanese population. Cross-ethnic validation may be needed, as genetic associations with AAM differ between Asian and European women [42]. Secondly, AAM and diagnosis of T2DM in TWB were based on self-reported questionnaires. Recall bias may exist. Thirdly, the use of hypoglycemic medications was not available in TWB. An augmented dataset linking the TWB and insurance claims of the National Health Insurance Research Database may help to clarify these issues. Fourthly, the GWAS results could be affected by selection bias, as individuals who volunteer to participate may not be representative of the underlying sampling population.

## 5. Conclusions

This study highlighted potential pleiotropic effects across AAM and T2DM that may explain the association between the two phenotypes. The result may suggest genetic screening for the enriched loci that may help to inform risks for T2DM in women with early AAM. The shared genetic variants discovered are targets for the future exploration of early AAM and T2DM mechanisms.

## Figures and Tables

**Table 1:** A total of 39 independent pleiotropic loci in AAM (trait 1) and T2DM (trait 2) with a conditional false discovery rate (cFDR) of less than 0.05. (Note: T2DM, type 2 diabetes mellitus; AAM, age at menarche; Chr, chromosome; hg38, Homo sapiens genome assembly GRCh38; BFP, body fat percentage; FBG, fasting blood glucose; HbA_1C_, glycated hemoglobin A1C).

Chr	rs	Position (hg38)	Gene	*p* of Trait 1	Trait 2	*p* of Trait 2	cFDR
1	rs148500298	1.02 × 10^8^	*RP11-202K23.1*	0.658659	FBG	5.25 × 10^−6^	0.040840737
1	rs3138105	1.58 × 10^8^	*CD1B*, *CD1C*	0.475425	HbA_1c_	9.86 × 10^−6^	0.045925272
1	rs17042165	2.15 × 10^8^	*LOC124904510*	0.00217096	HbA_1c_	4.61 × 10^−6^	0.007599174
2	rs780093	27,519,736	*GCKR*	0.320034	FBG	4.80 × 10^−7^	0.007515765
2	rs895636	44,961,214	*AC012354.6*	0.807344	FBG	1.67 × 10^−7^	0.003508871
2	rs1402837	1.69 × 10^8^	*G6PC2*	0.280055	HbA_1c_	1.44 × 10^−8^	0.00028397
					FBG	1.32 × 10^−13^	1.82 × 10^−8^
2	rs116933981	1.69 × 10^8^	*LRP2*	0.41804	FBG	8.63 × 10^−7^	0.012442801
3	rs3804766	51,393,226	*RBM15B*	0.208714	HbA_1C_	3.20 × 10^−6^	0.033487115
4	rs223461	1.03 × 10^8^	*LOC102723704, LOC124900743*	0.192597	HbA_1C_	5.45 × 10^−6^	0.048127897
4	rs79196252	1.47 × 10^8^	*SLC10A7*	0.0439132	FBG	8.66 × 10^−7^	0.010702327
5	rs248062	1.27 × 10^8^	*CTB-1I21.1*	0.0326756	BFP	4.10 × 10^−7^	0.007739594
5	rs75170429	1.33 × 10^8^	*FSTL4*	0.419274	HbA_1C_	2.51 × 10^−6^	0.019516832
6	rs2206734	20,694,653	*CDKAL1*	0.712727	HbA_1C_	1.94 × 10^−14^	6.49 × 10^−10^
					FBG	3.05 × 10^−7^	0.006010452
6	rs9376090	1.35 × 10^8^	*HBS1L*	0.695327	HbA_1C_	7.17 × 10^−6^	0.041947507
7	rs10244051	15,024,208	*GTF3AP5*	0.776841	FBG	8.57 × 10^−7^	0.012993208
7	rs16881016	44,171,808	*GCK*, *LOC105375257*	0.480132	HbA_1C_	4.52 × 10^−10^	8.01 × 10^−6^
					FBG	2.01 × 10^−11^	2.31 × 10^−6^
7	rs1799884	44,189,469	*GCK*	0.264629	FBG	3.12 × 10^−6^	0.029224933
7	rs6975024	44,192,287	*GCK*	0.601593	HbA_1C_	2.22 × 10^−7^	0.002111339
7	rs2233580	1.28 × 10^8^	*PAX4*	0.403983	T2DM	5.07 × 10^−10^	9.90 × 10^−5^
					HbA_1C_	6.18 × 10^−10^	1.51 × 10^−5^
					FBG	2.70 × 10^−7^	0.005270602
7	rs6943771	1.4 × 10^8^	*PARP12*	0.995326	FBG	4.37 × 10^−6^	0.038811336
8	rs3802177	1.17 × 10^8^	*SLC30A8*, *LOC105375716*	0.889634	HbA_1C_	8.07 × 10^−12^	1.67 × 10^−7^
					FBG	7.81 × 10^−12^	1.62 × 10^−6^
9	rs10965250	22,133,285	*CDKN2B-AS1*	0.236788	HbA_1C_	8.65 × 10^−13^	1.02 × 10^−7^
					FBG	3.00 × 10^−11^	3.54 × 10^−6^
9	rs1050700	1.33 × 10^8^	*TSC1*	0.487359	HbA_1C_	1.16 × 10^−5^	0.049462307
10	rs12221133	12,211,598	*CDC123*	0.45323	HbA_1C_	1.44 × 10^−6^	0.010460355
10	rs151268010	16,675,558	*RSU1*	0.0122221	BFP	3.57 × 10^−6^	0.027178562
10	rs75631171	92,449,649	*IDE*	0.190513	T2DM	2.55 × 10^−7^	0.024501519
10	rs1111875	92,703,125	*Y_RNA*	0.664192	T2DM	2.59 × 10^−7^	0.027071426
10	rs10786156	94,254,865	*PLCE1*	0.332509	BFP	1.02 × 10^−8^	0.00165048
11	rs60808706	2,836,003	*KCNQ1*	0.589924	HbA_1C_	3.38 × 10^−12^	9.49 × 10^−8^
					FBG	6.33 × 10^−9^	0.000221717
11	rs10466351	92,964,815	*LOC124902733*	0.44799	FBG	1.25 × 10^−10^	6.76 × 10^−6^
11	rs10830963	92,975,544	*MTNR1B*	0.526219	HbA_1C_	6.32 × 10^−7^	0.005298336
17	rs761772	78,125,997	*TMC6*	0.429945	HbA_1C_	4.79 × 10^−12^	1.42 × 10^−7^
17	rs73357173	82,670,096	*RAB40B/MIR4525*	0.0342794	HbA_1C_	2.89 × 10^−6^	0.019044672
17	rs2250754	82,703,440	*LOC124904093*	0.0102342	HbA_1C_	1.73 × 10^−6^	0.01124699
17	rs1046896	82,727,657	*FN3KRP*	0.382707	HbA_1C_	3.68 × 10^−54^	6.84 × 10^−49^
17	rs3785519	82,937,832	*TBCD*	0.177841	HbA_1C_	2.08 × 10^−8^	0.000467929
17	rs58431774	83,000,587	*B3GNTL1*	0.742557	HbA_1C_	7.61 × 10^−15^	2.95 × 10^−10^
21	rs7510550	36,629,993	*AP000696.2*	0.515658	FBG	7.92 × 10^−7^	0.01085386
22	rs146847831	48,773,800	*FAM19A5*	0.106661	FBG	1.42 × 10^−6^	0.019983018

## Data Availability

The TWB dataset is available upon application for research purposes. A detailed description of TWB data availability and the application process could be found via the following link: https://taiwanview.twbiobank.org.tw/data_appl.

## Supplementary Materials

The following supporting information can be downloaded via this link: https://www.mdpi.com/article/10.3390/biomedicines12010157/s1, Figure S1: Description of quality control procedures; Figure S2: Risk of T2DM based on age of participants; Figure S3: Protein-protein interaction (PPI) network for novel pleiotropic loci; Table S1: Description of quality control procedures; Table S2: General characteristics of participants in 2 datasets; Table S3: Top 10 enriched Kyoto Encyclopedia of Genes and Genomes (KEGG) pathways.
