# Supplementary material for: Shared Genetics between Age at Menarche and Type 2 Diabetes Mellitus: Genome-Wide Genetic Correlation Study
Source: Biomedicines. 2024 Jan 11;12(1):157. doi: 10.3390/biomedicines12010157 (PMC10813301; doi:10.3390/biomedicines12010157)
Supplement: Supplementary file 1 [file biomedicines-12-00157-s001.zip › biomedicines-2790406-supplementary.pdf]

Supplementary Information

PLINK functions	Meaning
--filter-males	filters out males
--geno 0.02	filters out SNPs with genotype missing rate higher than 2%
--mind 0.02	filters out samples with genotype missing rate higher that 2%
--maf 0.01	filters out SNPs with minor allele frequency (MAF)* below 0.01
--hwe 0.0000001	filters out SNPs which have Hardy-Weinberg equilibrium* exact test p-value below 1E-06
--prune	filters out samples with missing phenotypes

Table S1. Description of quality control procedures

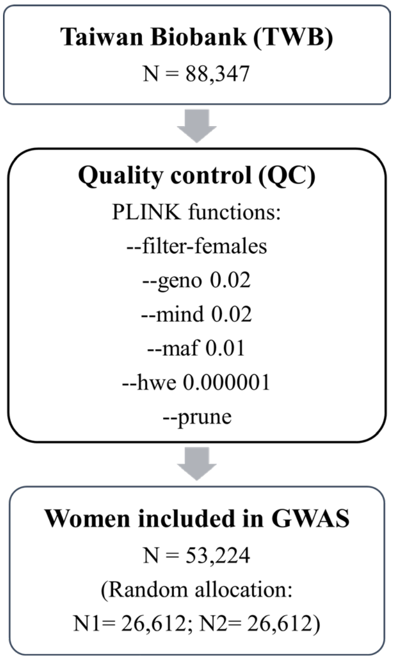

Figure S1. Description of quality control procedures

*Minor allele frequency (MAF): The frequency at which a less common allele occurs in a population. SNPs with very low MAF are prone to genotyping errors.

*Hardy-Weinberg equilibrium (HWE): The frequency of genotypes in a population may be calculated using the formula $p^2 + q^2 + 2pq = 1$. SNPs deviating from HWE are prone to genotyping errors.

Table S2. General characteristics of participants in 2 datasets

**Characteristics**		**GWAS of AAM (N = 26612)**		**GWAS of T2DM (N = 26612)**	
		N or mean	% or SD	N or mean	% or SD
**Education level**					
Low		1509	5.67	1572	5.9
Medium		10537	39.59	10686	40.2
High		14566	54.73	14354	53.9
**Age**					
Year		50.82	10.77	51.00	10.72
Older (≥ 65)		2750	10.33	2771	10.41
**AAM**					
Year		13.26	1.47	13.26	1.47
Early (≤ 11)		2918	10.96	2917	10.96
**Diabetic factors**					
T2DM	Yes	1158	4.351	1164	4.374
BMI	$kg \cdot m^{-2}$	23.63	3.80	23.62	3.74
BMI ≥ 24.5	Yes	9055	34.03	9171	34.46
Waistline	cm	80.79	9.75	80.74	9.71
Waistline ≥ 80	Yes	13572	51.00	13481	50.66
BFR	%	32.10	6.40	32.08	6.37
BFR ≥ 30	Yes	16508	62.03	16508	62.03
FBG	$mg \cdot dl^{-1}$	94.22	18.43	94.41	18.85
HbA_{1c}	%	5.76	0.73	5.77	0.75
SBP	mmHg	117.73	18.09	118.05	18.48
DBP	mmHg	71.16	10.30	71.26	10.44
TG	$mg \cdot dl^{-1}$	105.40	77.35	104.97	73.95
Total cholesterol	$mg \cdot dl^{-1}$	198.93	36.34	198.92	36.14
HDL-C	$mg \cdot dl^{-1}$	58.49	13.37	58.44	13.37
LDL-C	$mg \cdot dl^{-1}$	120.83	31.83	120.89	32.06

Abbreviations: GWAS, genome-wide association study; N, total number; SD, standard deviation; T2DM,

type 2 diabetes mellitus; BMI, body mass index; BFR, body fat rate; FBG, fasting blood glucose; HbA_{1c}, glycated hemoglobin A1C; SBP, systolic blood pressure; DBP, diastolic blood pressure; TG, triglyceride; HDL-C, high-density lipoprotein cholesterol; LDL-C, low-density lipoprotein cholesterol.

AAM (trait 1)

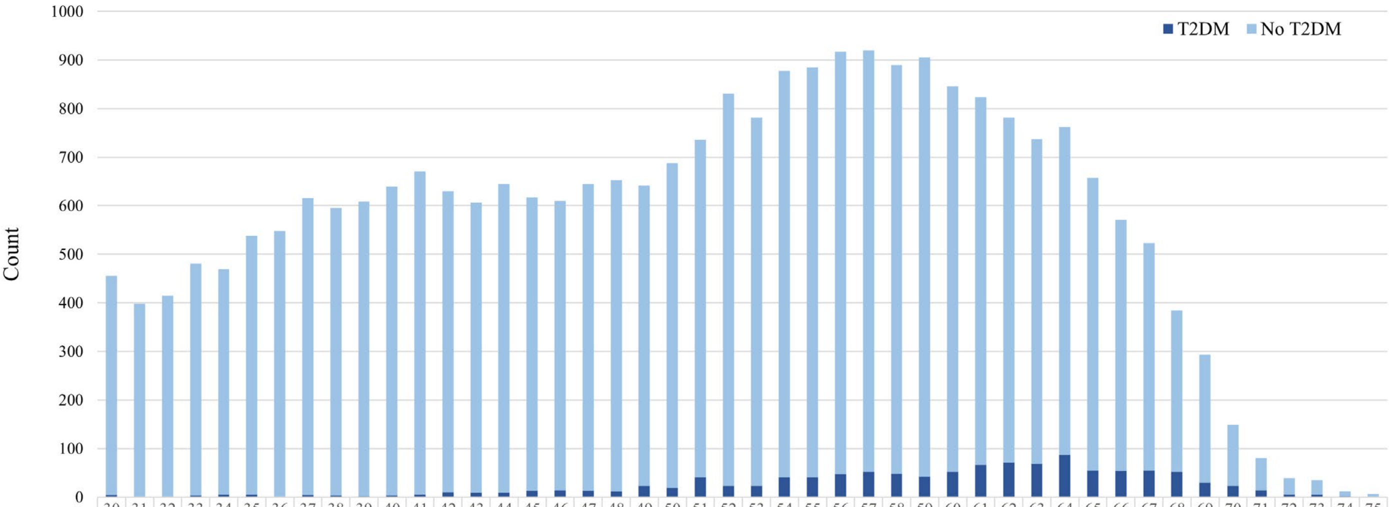

Age	30	31	32	33	34	35	36	37	38	39	40	41	42	43	44	45	46	47	48	49	50	51	52
No T2DM	452	397	414	478	464	533	547	612	592	606	637	665	620	598	636	604	596	632	641	618	669	695	808
T2DM	4	1	1	3	5	5	1	4	3	2	3	5	10	9	9	13	14	13	12	23	19	41	23

Age	53	54	55	56	57	58	59	60	61	62	63	64	65	66	67	68	69	70	71	72	73	74	75
No T2DM	758	837	844	870	868	842	863	794	757	710	668	675	602	517	468	332	263	126	66	34	30	10	6
T2DM	23	41	41	47	52	48	42	52	66	71	69	87	55	54	55	52	30	23	14	5	5	2	1

Age

T2DM and relevant phenotypes (trait 2)

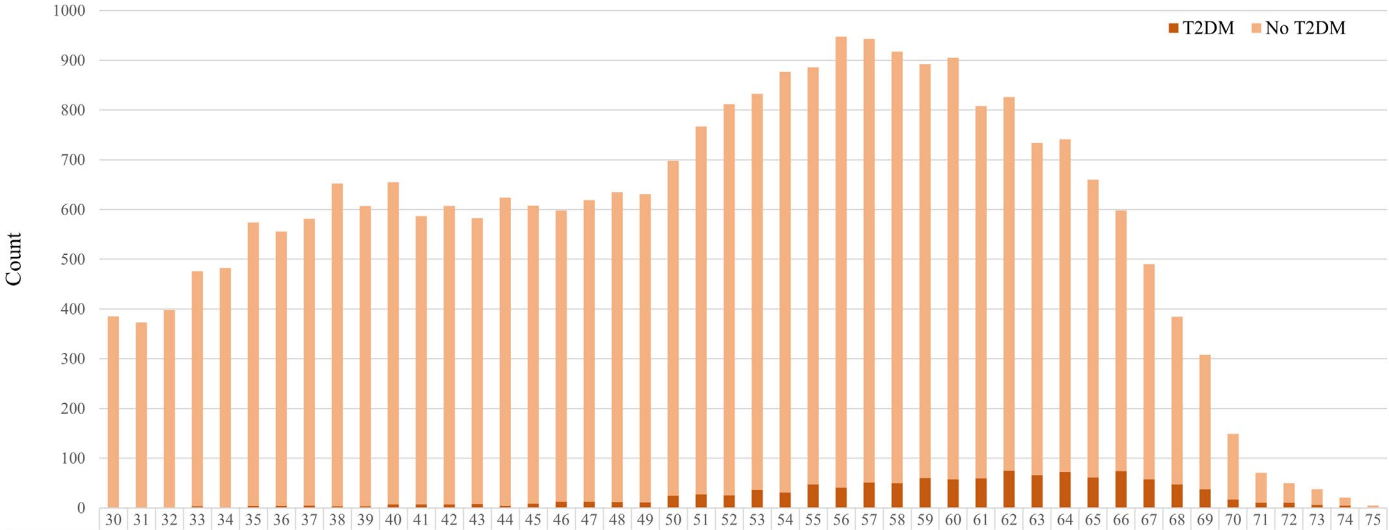

Age	30	31	32	33	34	35	36	37	38	39	40	41	42	43	44	45	46	47	48	49	50	51	52
No T2DM	384	373	398	473	482	570	551	576	649	604	648	580	600	575	620	599	585	606	623	620	673	740	786
T2DM	1	0	0	3	0	4	4	5	3	3	7	7	7	8	4	9	13	13	12	11	25	27	26

Age	53	54	55	56	57	58	59	60	61	62	63	64	65	66	67	68	69	70	71	72	73	74	75
No T2DM	797	846	839	907	892	867	832	847	749	751	668	669	599	524	432	337	270	132	61	40	32	16	5
T2DM	36	31	47	41	51	50	60	58	59	75	66	72	61	74	58	47	38	17	10	10	6	5	0

Age

Figure S2. Risk of T2DM based on age of participants

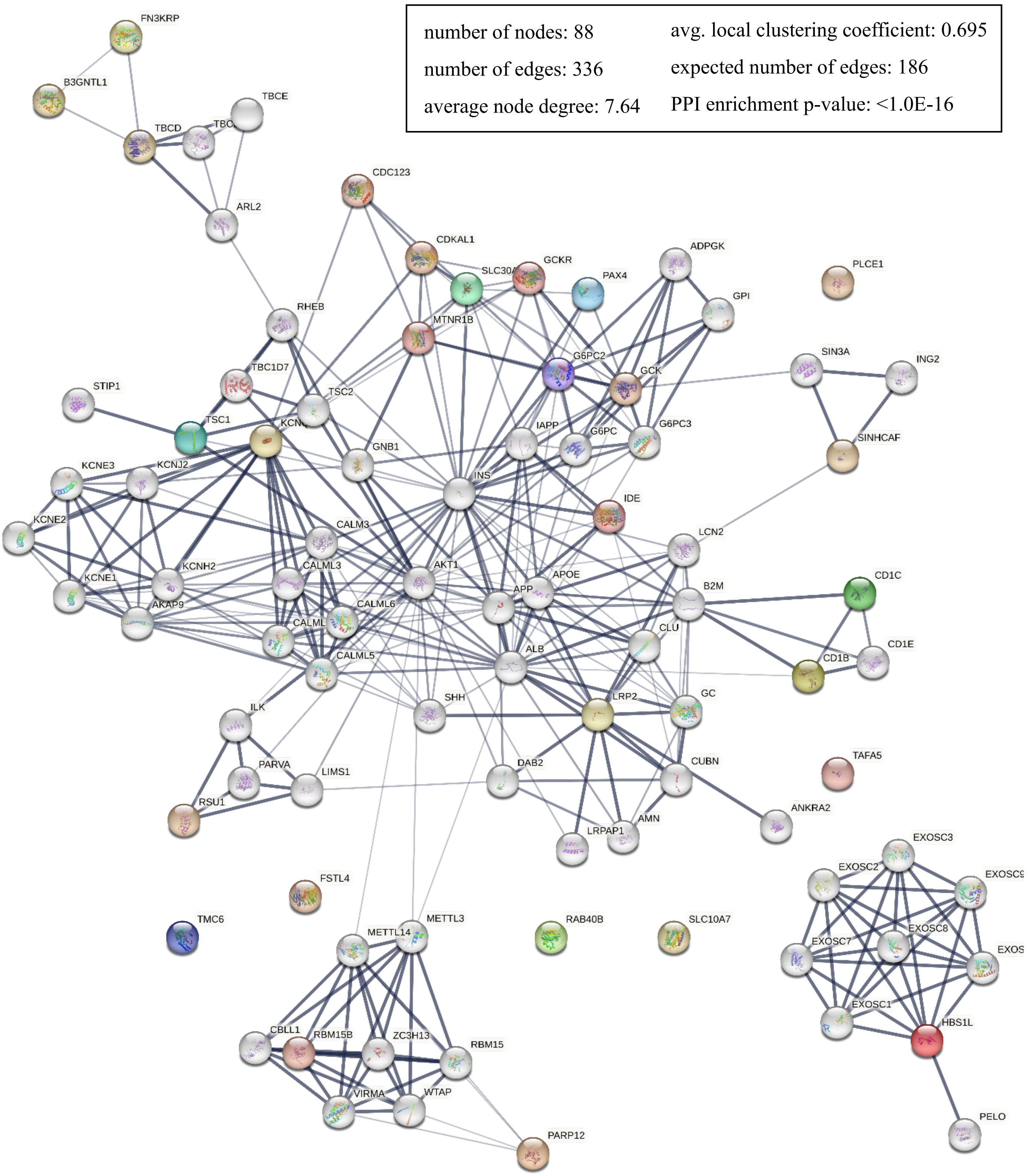

	Meaning	Characteristics	Implication
**nodes**	proteins	colored	query proteins
		white	other interactor proteins
		empty	proteins of unknown 3D structure
		filled	some 3D structure is known or predicted
**edges**	protein-protein associations	line thickness	the strength of data support

Figure S3. Protein-protein interaction (PPI) network for novel pleiotropic loci

Table S3. Top 10 enriched Kyoto Encyclopedia of Genes and Genomes (KEGG) pathways

Pathway	False discovery rate
Insulin signaling pathway	1.02e-12
Glucagon signaling pathway	9.81e-09
Gastric acid secretion	2.5e-07
Phototransduction	4.51e-07
Human cytomegalovirus infection	4.51e-07
Cellular senescence	2.07e-06
RNA degradation	3.76e-06
AMPK signaling pathway	4.30e-06
Circadian entrainment	1.01e-05
Adrenergic signaling in cardiomyocytes	1.43e-05
